# Bleomycin Electrosclerotherapy (BEST) for Slow-Flow Malformations of the Upper Aerodigestive Tract

**DOI:** 10.3390/biomedicines13051055

**Published:** 2025-04-27

**Authors:** Veronika Vielsmeier, Vanessa F. Schmidt, Florian Obereisenbuchner, Natascha Platz Batista da Silva, Walter A. Wohlgemuth, Daniel Puhr-Westerheide, Max Seidensticker, Jens Ricke, Thomas Kühnel, Christopher Bohr, Moritz Wildgruber, Caroline T. Seebauer

**Affiliations:** 1 Department of Otorhinolaryngology, Regensburg University Medical Center, 93053 Regensburg, Germany; veronika.vielsmeier@ukr.de (V.V.); thomas.kuehnel@ukr.de (T.K.); christopher.bohr@ukr.de (C.B.); 2 Department of Radiology, LMU University Hospital, LMU Munich, 81377 München, Germany; vanessa.schmidt@med.uni-muenchen.de (V.F.S.); florian.obereisenbuchner@med.uni-muenchen.de (F.O.); daniel.puhr-westerheide@med.uni-muenchen.de (D.P.-W.); max.seidensticker@med.uni-muenchen.de (M.S.); jens.ricke@med.uni-muenchen.de (J.R.); moritz.wildgruber@med.uni-muenchen.de (M.W.); 3 Institute of Radiology, Regensburg University Medical Center, 93053 Regensburg, Germany; natascha.platz-batista-da-silva@ukr.de; 4 Department of Radiology and Policlinic of Radiology, University Hospital Halle (Saale), 06120 Halle (Saale), Germany; walter.wohlgemuth@uk-halle.de; 5 Department of Otorhinolaryngology, Head and Neck Surgery, Luzerner Kantonsspital, 6000 Lucerne, Switzerland

**Keywords:** slow-flow malformation, venous malformation, lymphatic malformation, electrosclerotherapy, BEST, bleomycin, mucosal malformation, sclerotherapy, vascular anomalies, vascular malformations

## Abstract

**Background/Objectives:** Bleomycin electrosclerotherapy (BEST), which combines intralesional bleomycin administration with electroporation, enhances drug uptake and has shown efficacy in treating vascular malformations resistant to conventional therapies. While BEST is increasingly used in various anatomical sites, its application in the upper aerodigestive tract remains underexplored. This study evaluates the safety and effectiveness of BEST in managing slow-flow vascular malformations of the oral cavity, tongue, larynx, and hypopharynx. **Methods:** In this retrospective, multicenter study, 20 patients with symptomatic slow-flow vascular malformations of the upper aerodigestive tract were treated with BEST. Clinical and radiological assessments were used to evaluate the treatment response, categorized as “significantly reduced”, “reduced”, “stable disease”, or “lesion growth”. Postprocedural complications and functional outcomes were systematically recorded. **Results:** A total of 29 BEST sessions were performed. Lesions of the tongue (*n* = 8) and combined oral cavity and tongue (*n* = 6) showed the highest response rates, with significant symptom reduction in five out of eight and five out of six patients, respectively. Among isolated oral cavity lesions (*n* = 4), one out of four demonstrated a significant reduction. In contrast, laryngeal and hypopharyngeal lesions (*n* = 2) had limited response, with one case showing partial reduction and the other remaining stable. Severe complications, including bleeding and dyspnea requiring tracheostomy, limited further treatment in these locations. No systemic adverse events, such as pulmonary toxicity, were observed. **Conclusions:** BEST is effective for treating vascular malformations of the upper aerodigestive tract, particularly in the tongue and oral cavity, but presents significant risks in laryngeal and hypopharyngeal lesions. A multidisciplinary approach is required to optimize treatment protocols for these challenging locations.

## 1. Introduction

Bleomycin electrosclerotherapy (BEST) represents an innovative approach in the treatment of vascular malformations, particularly for cases resistant to conventional therapies [1]. Slow-flow vascular malformations of the head and neck are challenging due to the wide range of clinical presentations from esthetic concerns to functional impairment, or even life-threatening complications. Conventional treatments, such as surgery and sclerotherapy, have shown limitations in achieving complete and sustained resolution, particularly in complex anatomic locations along the upper aerodigestive tract. Often, multiple treatments are required to achieve satisfactory results. The administration of bleomycin, a cytotoxic antibiotic with sclerosing properties, in combination with reversible electroporation, a technique involving the application of electric pulses to enhance drug delivery by increasing cell membrane permeability and inducing targeted sclerosis, obliterates the anomalous vascular tissue while minimizing systemic side effects [2].

The first documented case of BEST was reported by McMorrow et al., who successfully treated a patient with a venous malformation and severe respiratory compromise [1]. This case illustrated the potential of BEST to achieve effective results using lower doses of bleomycin, thus minimizing systemic risks such as pulmonary fibrosis. Electroporation augmented the local delivery of bleomycin, enabling the effective management of the malformation while addressing the patient’s respiratory concerns.

Wohlgemuth et al. provided evidence supporting the effectiveness of BEST for therapy-resistant venous malformations. Their retrospective observational study revealed an 86% reduction in lesion volume and substantial symptomatic improvement in the majority of patients after treatment [3]. Further studies highlighted the effectiveness of BEST in treating vascular malformations, emphasizing its potential for improved outcomes in cases where other treatments have failed [4]. So far, the literature on the application of this treatment modality in the head and neck region is limited. Therefore, we investigated the effectiveness and safety of BEST on the mucosa of the aerodigestive tract in a multicenter cohort of patients.

## 2. Methods

### 2.1. Study Design

This retrospective, multicenter study included patients with symptomatic slow-flow vascular malformations (both simple and combined), classified according to the International Society for the Study of Vascular Anomalies (ISSVA) criteria, treated with bleomycin electrosclerotherapy (BEST) between January 2022 and February 2024. This study was approved by the local ethics committee (University of Regensburg, Protocol No. 17-854_1-101 and LMU University Hospital, Protocol No. 23-0035) and conducted in accordance with the principles of the 1964 Helsinki Declaration and its subsequent amendments. Data were collected using electronic patient records and the Picture Archiving and Communication System (PACS). Diagnosis and therapeutic indications for electrosclerotherapy were established through an interdisciplinary consensus process based on patient history, ultrasonography, magnetic resonance imaging (MRI), and clinical examination. Biopsies with histopathological assessment were performed only when a definitive diagnosis could not be established through these methods. Decisions regarding BEST were made at two specialized interdisciplinary vascular anomalies centers in Germany, involving at least one interventional radiologist, head and neck surgeon, and, for patients younger than 18 years, a pediatrician or pediatric surgeon. Additional specialties, such as hematology–oncology, maxillofacial surgery, plastic surgery, and hemostaseology, were consulted as needed. Indications for BEST included swelling, esthetic disfigurement, pain, bleeding, recurrent thrombosis, recurring infections, and functional impairments such as dysphagia, dyspnea, and speech impairment. Patients with prior interventional or surgical treatments were included if there was a therapy-free interval of at least 6 months before their first BEST procedure. BEST was generally contraindicated in breastfeeding or pregnant women, patients of childbearing potential not using effective contraception, individuals with bleomycin intolerance or previous bleomycin-related toxicity, those who had received a cumulative bleomycin dose of ≥100 mg, patients with chronic pulmonary dysfunction or prior chest radiation therapy, and individuals with a history of epilepsy or seizures.

### 2.2. Interventional Treatment

BEST procedures were conducted under general anesthesia following current operating procedures [5]. For complex lesions or cases with uncertain venous drainage patterns, a direct percutaneous injection of a contrast agent into the malformation was performed under fluoroscopic guidance. Following either the intravenous or intralesional administration of bleomycin, electrodes were positioned, and reversible electroporation pulses were subsequently applied. Electrodes were selected based on the lesion’s size, exposure requirements, and anatomical location. The options included the finger electrodes (IGEA S.p.A., Carpi, Italy, F-15-NO, IG0E375, 15 mm, orthogonal and F-10-NL, IG0E370, 10 mm, longitudinal) and the Stinger electrodes (IGEA S.p.A., Carpi, Italy, E-L5-00-S4-2, IG0E805, laparoscopic electrode, non-divergent, Ø 5 mm, active length of 20 mm, exposure range of 0–40 mm). Whenever technically feasible, the entire target volume was treated through repetitive punctures, ensuring uniform coverage and avoiding significant gaps or overlaps. The maximum overall dose of bleomycin allowed per session (intralesional and/or intravenous) was 0.2 mg/kg body weight, with a cumulative dose capped at less than 1 mg/kg body weight, adhering to standard bleomycin sclerotherapy protocols and remaining lower than previously reported doses for cutaneous tumors and skin metastases [6,7]. Reversible electric pulses were delivered using the Cliniporator™ VITAE electroporation system (IGEA S.p.A., Carpi, Italy), which features multiple independently controlled and isolated outputs capable of generating up to 3000 V/cm (maximum current: 50 A). Electrical pulses of 100 μs duration were applied between each electrode pair. Electroporation was performed immediately after intralesional bleomycin administration or 8 min after the initiation of intravenous bleomycin application.

### 2.3. Follow-Up

At both participating centers, patients were enrolled in a standardized follow-up protocol. The first clinical follow-up was conducted 90 days (range: 34–392 days) after the first BEST session. If symptom improvement was insufficient and residually perfused lesions were identified, additional BEST sessions were scheduled. If the initial BEST indicated no therapeutic response, no subsequent treatments were scheduled. During follow-up visits, lesion regression was assessed by two vascular anomalies specialists. Postinterventional lesion outcomes were graded by comparing pre- and postinterventional photographic documentation, MRI, or a combination of both. Outcomes were categorized as “reduced lesion”, “significantly reduced lesion”, “stable disease”, or “lesion growth”. A lesion was classified as “reduced” if a measurable decrease in size was observed clinically or via imaging, while “significantly reduced” was defined as a lesion that became either invisible or barely detectable. “Stable disease” indicated no change. An increase in lesion size was classified as “lesion growth”. The classification was performed independently by two physicians. Patients were systematically queried about postprocedural swelling, pain, esthetic concerns, functional impairments, and bleeding. A comprehensive clinical examination was performed by a specialist, focusing on lesion size, swelling, thrombophlebitis, infections, and functional outcomes related to dysphagia, dyspnea, and impaired speech.

### 2.4. Statistical Analysis

Descriptive statistics were employed to analyze the distribution of patients across different categories. The Kolmogorov–Smirnov (K–S) test was applied to assess the normality of data. Results are presented as mean ± standard deviation for normally distributed data or as median (range: minimum–maximum) for non-normally distributed data. Subgroup analyses for categorical variables were conducted using Pearson’s chi-squared test. Statistical analyses were performed with GraphPad Prism software, version 9.0. Figure legends provide details on sample sizes and significance levels. A *p*-value < 0.05 was considered statistically significant.

## 3. Results

### 3.1. Patient Characteristics

A total of twenty patients, seven males and thirteen females, with symptomatic, extracranial, slow-flow vascular malformations involving the mucosa of the aerodigestive tract underwent BEST treatment (Table 1). The median age at the time of the first treatment was 39 years (range: 4–78 years). In terms of lesion type, fifteen patients presented with simple venous malformations (VMs), three patients with simple lymphatic malformations (LMs), and two patients with combined veno-lymphatic malformations (VLMs). The anatomical distribution of mucosal involvement in the head and neck region was as follows: the laryngeal and hypopharyngeal mucosa in two patients, the mucosa of the tongue alone in eight patients, the mucosa of the oral cavity alone in four patients, and the mucosa of both the oral cavity and the tongue in six patients. The study included both treatment-naive patients (12/20) and those with a history of prior invasive treatments (8/20), comprising debulking surgery (two patients), laser therapy (one patient), sclerotherapy (four patients), or combined therapy involving surgery, sclerotherapy, and sirolimus (one patient). All previously treated patients experienced insufficient symptom improvement prior to inclusion in this study. Further, there was a therapy-free interval of at least 6 months before their first BEST procedure.

### 3.2. Procedural Characteristics

**Figure 1:**
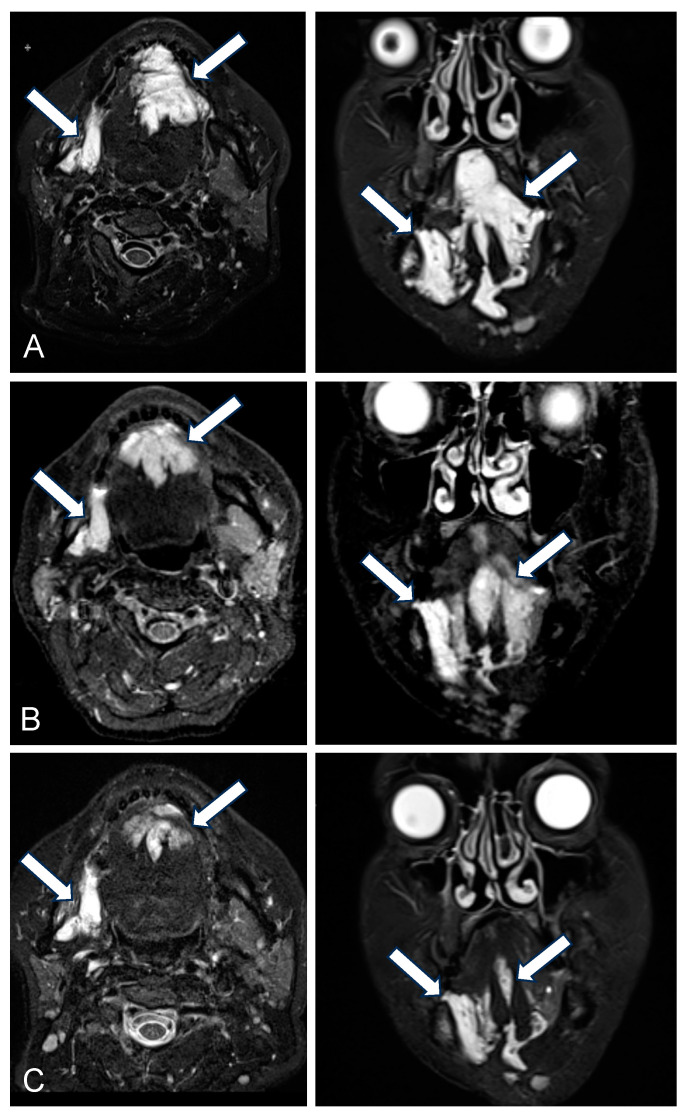
A 49-year-old female patient with an extensive venous malformation (VM, indicated by white arrows) of the oral cavity and tongue undergoing 3 sequential bleomycin electrosclerotherapy (BEST) sessions with intralesional drug administration. (**A**) T2-weighted (T2w) axial and coronal magnetic resonance (MR) images obtained prior to BEST treatment, with indications including swelling, pain, dysphagia, and impaired speech. (**B**) T2w axial and coronal MR images acquired 3 months following the second BEST session, demonstrating partial regression of the malformation. (**C**) T2w axial and coronal MR images 6 months after the third BEST session, showing near-complete resolution of the malformation. Following the third session, no further swelling or pain was reported, and lesion regression led to the restoration of unimpaired food intake and speech.

A total of twenty patients with mucosal involvement in the head and neck region underwent 29 BEST treatments, with thirteen patients undergoing a single procedure, five patients undergoing 2 procedures, and two patients undergoing 3 procedures, resulting in a mean of 1.5 ± 0.7 procedures per patient (Table 2). All treatments were conducted under general anesthesia. Of the 29 procedures, 8 were performed on pediatric patients (<18 years). Orthogonal 15 mm finger electrodes (F-15-NO, IGEA S.p.A., Carpi, Italy) were predominantly used in 21 of 29 procedures, while 10 mm longitudinal finger electrodes (F-10-NL, IGEA S.p.A., Carpi, Italy) and non-divergent Stinger electrodes (E-L5-00-S4-2, IGEA S.p.A., Carpi, Italy) were utilized in 6 and 2 procedures. The mean number of electroporation cycles per treatment was 12.7 ± 13.7. Bleomycin was administered intralesionally in 28 of 29 cases with a mean dose of 5.7 ± 4.2 mg per session (Figure 1). In one case, the systemic administration of bleomycin was performed at a dose of 7 mg intravenously due to the diffuse distribution of the lesion within the oral cavity and tongue, as a direct puncture of the entire lesion was not feasible. The mean duration of the procedure was 66.2 ± 40.2 min. Procedures were carried out by an otorhinolaryngologist in six cases, an interventional radiologist in fourteen cases, and through a combined approach in nine cases. Prophylactic anticoagulation with enoxaparin was administered to 16 patients in the postprocedural course. Patients had an average hospital stay of 7.0 ± 7.1 days, and 11 patients required postprocedural monitoring in the intensive care unit (ICU), with a mean ICU stay of 5.4 ± 6.4 days.

### 3.3. Effectiveness of Treatment

**Figure 2:**
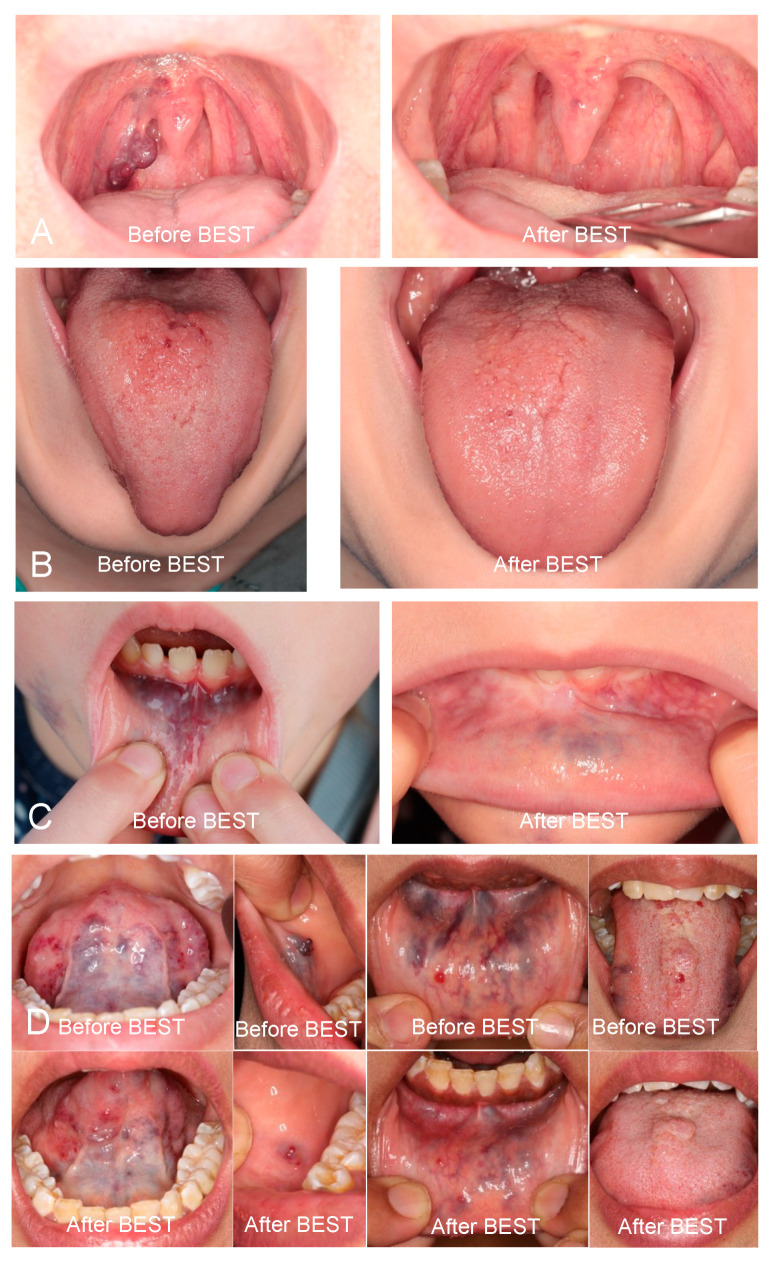
Patients with lesions in the oral cavity and tongue demonstrated the highest rates of lesion reduction following bleomycin electrosclerotherapy (BEST). (**A**) A 41-year-old male patient with a venous malformation (VM) of the right palatal arch presented with swelling, dysphagia, and dyspnea. Three months post-treatment, the lesion was completely resolved, and the patient was symptom-free. (**B**) A 10-year-old male patient with a lymphatic malformation (LM) of the tongue presented with swelling, pain, and dysphagia. Seven months post-treatment, the lesion was completely resolved, and the patient was symptom-free. (**C**) A 6-year-old female patient with a VM of the lower lip mucosa presented with swelling, pain, and dysphagia. One year post-treatment, the lesion was significantly reduced, and the patient was symptom-free. (**D**) A 26-year-old female patient with a VM involving the floor of the mouth, tongue, mucosa of the right cheek, and lower lip presented with swelling and pain. Three months post-treatment, the lesion was significantly reduced, and the patient was symptom-free.

**Figure 3:**
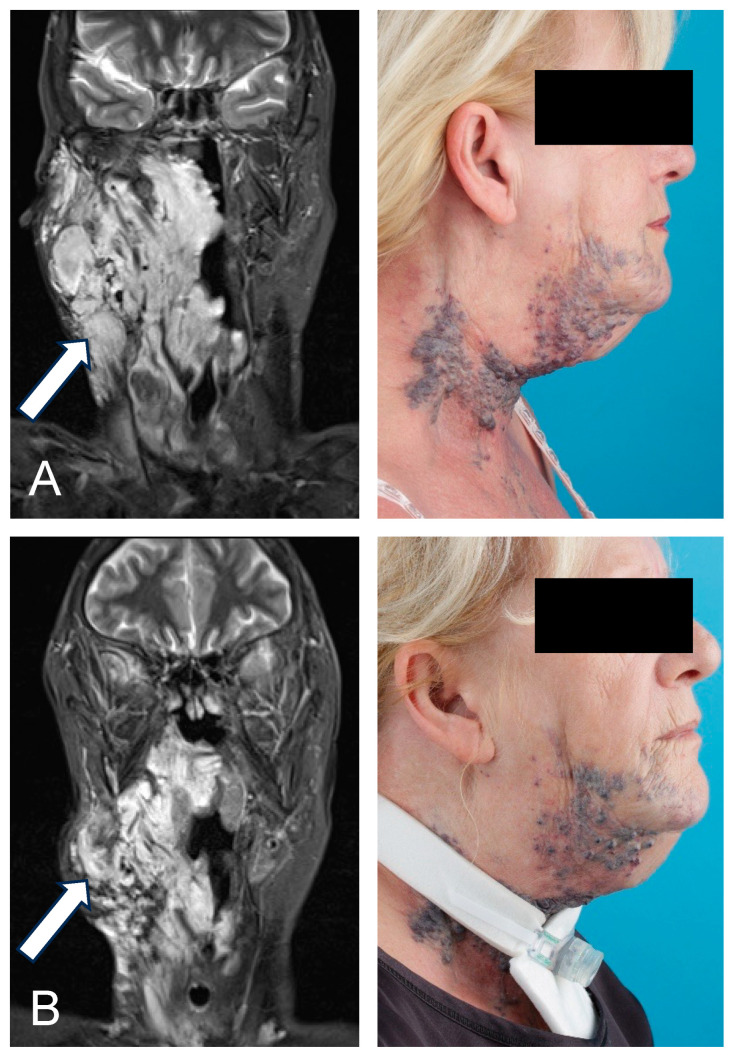
A 64-year-old female patient with an extensive venous malformation (VM, indicated by arrows) of the laryngeal and hypopharyngeal mucosa with severe bleeding, swelling, and dyspnea following bleomycin electrosclerotherapy (BEST). (**A**) T2-weighted (T2w) coronal magnetic resonance (MR) image and sagittal photographic documentation of the lesion prior to treatment, showing the extent of the VM. The patient reported symptoms of dysphagia and dyspnea. (**B**) T2w coronal MR image and sagittal photographic documentation taken 2 months after the first BEST session demonstrated regression of the lesion. However, postinterventional swelling and bleeding necessitated a tracheostomy, precluding a second BEST procedure.

To evaluate treatment effectiveness, outcomes were categorized into groups: “reduced lesion”, “significantly reduced lesion”, “stable disease”, or “lesion growth”. The median follow-up after the last treatment in the cohort was 100 days (range: 5–392 days). An increase in lesion size classified as “lesion growth” was not observed in the cohort. Overall, 8/20 patients showed partial lesion size reduction, 11/20 exhibited significant lesion size reduction, and in 1/20, stable disease was observed (Table 3). Response rates varied significantly by anatomical location (Pearson’s chi-squared test, *p* = 0.0318). Patients with lesions in the tongue (5/8) and the oral cavity and tongue (5/6) exhibited the highest rates of significant reduction, respectively (Figure 2 and Supporting Video S1A–C). In the oral cavity, 3/4 of patients achieved partial reduction, and 1/4 of patients demonstrated significant reduction. In contrast, lesions in the laryngeal and hypopharyngeal mucosa showed limited response, with 1/2 of patients achieving partial reduction and 1/2 of patients remaining stable without improvement (Figure 3 and Supporting Video S2A–C).

### 3.4. Functional Outcomes and Complications

A reduction in symptoms was observed across all anatomical locations following bleomycin electrosclerotherapy (BEST) (Table 4). Dysphagia showed improvement, decreasing from 15 symptomatic patients (15/20) before treatment to 3 (3/20) after the first round of treatment. Excluding cases affecting the laryngeal and hypopharyngeal mucosa (2/20), dysphagia completely resolved after the third treatment. Swelling was notably reduced across most locations, decreasing from 16 symptomatic patients (16/20) before treatment to a lower incidence (3/20) after the first treatment. However, these changes did not reach statistical significance. Improvements were particularly pronounced in the tongue and oral cavity regions, where swelling and pain symptoms resolved completely in all affected patients. Swelling, which affected 7/8 patients with tongue involvement before treatment, was reduced to 1/8 patients after the first BEST and was entirely absent after the second BEST. Pain symptoms, present in 5/8 patients in the same group before treatment, were also fully resolved. In the oral cavity and tongue group, speech impairment, pain, bleeding, and recurrent infections showed complete resolution following treatment, reflecting the highest improvements observed across the cohort. Patients with oral cavity involvement demonstrated improvements in cosmetic concerns and pain symptoms, with esthetic concerns reducing from 2/4 symptomatic patients before treatment to none post-treatment. In contrast, patients with laryngeal and hypopharyngeal mucosa involvement exhibited more limited responses, with dysphagia and dyspnea persisting in 1/2 of cases. Furthermore, these anatomical regions were associated with severe procedural complications, such as bleeding and swelling, resulting in a tracheostomy (Figure 3). Consequently, no further patients with slow-flow lesions of the laryngeal or hypopharyngeal mucosa were treated with BEST.

Postinterventional aggravation of symptoms was observed in 17/20 of patients following the first BEST treatment. Postinterventional symptoms varied across anatomical locations, with the highest prevalence in patients with laryngeal and hypopharyngeal mucosa (2/2) and oral cavity and tongue involvement (6/6), followed by tongue (6/8) and oral cavity (3/4). The most frequently reported complication was swelling, affecting 14/20 patients. Swelling was most prevalent in patients with oral cavity and tongue involvement (5/6) and tongue lesions (6/8). Pain was reported in 3/20 patients, primarily in the oral cavity group (2/4) and tongue group (1/8). Bleeding occurred in 4/20 patients, with contributions from all anatomical groups, including laryngeal and hypopharyngeal mucosa (1/2), tongue (1/8), oral cavity and tongue (1/6), and oral cavity (1/4). Dysphagia was reported in 6/20 patients. Infections were rare, affecting only 1/20 patients, exclusively in the oral cavity group. The most severe complication, dyspnea requiring tracheostomy or temporary protective intubation, was observed in 4/20 patients, exclusively in the laryngeal and hypopharyngeal mucosa (2/2) and tongue (2/8) groups. No systemic complications, such as pulmonary toxicity, were reported. Most importantly, all postinterventional symptoms of the tongue and oral cavity regions resolved spontaneously or were alleviated after a second or third treatment cycle with BEST.

These findings underscore the procedural challenges associated with BEST, particularly in anatomical locations such as the laryngeal and hypopharyngeal mucosa. Swelling and bleeding were the most common complications across all locations, whereas dyspnea with the need for tracheostomy and infections were rare but severe.

## 4. Discussion

Bleomycin electrosclerotherapy has emerged as a promising therapeutic modality for vascular malformations, particularly for slow-flow lesions that are resistant to conventional approaches, such as sclerotherapy or surgery [3,8]. The results of our study align with previous findings, highlighting the effectiveness of BEST in reducing lesion volume and alleviating symptoms, especially in complex anatomical regions in both children and adults [9,10,11]. Guntau et al. reported significant volume reduction and improved symptoms following BEST in patients with vascular malformations of the tongue, emphasizing the potential for targeted electroporation to enhance bleomycin delivery and therapeutic outcomes in the aerodigestive tract [12].

While BEST has shown significant effectiveness, its outcomes appear influenced by lesion location and complexity. In our study, complications such as persistent dysphagia and dyspnea were more prevalent in the laryngeal and hypopharyngeal mucosa group, emphasizing the challenges associated with treating vascular malformations in anatomically sensitive regions. These complications may stem from the highly vascularized and functionally critical nature of the laryngeal and hypopharyngeal mucosa, where tissue swelling, bleeding, and increased permeability induced by electroporation may result in rapid airway compromise. Furthermore, the close proximity of these lesions to vital airway structures likely exacerbates the risk of post-treatment obstruction, even when the local reaction is modest. Others reported similar challenges in treating LMs of the tongue, where interdisciplinary approaches combining BEST with surgical intervention were necessary to optimize functional outcomes [13]. In our cohort, lesions of the tongue and oral cavity demonstrated a particularly favorable response to the treatment. However, lesions of the laryngeal and hypopharyngeal mucosa did not regress to the same extent. Moreover, the occurrence of life-threatening complications, such as bleeding and swelling resulting in tracheostomy, precluded the continuation of BEST treatment in this region. To mitigate these risks, potential strategies may include pretreatment imaging to better characterize lesion depth and vascularity, real-time airway monitoring, and lower energy electroporation protocols tailored for mucosal tissue. Future technical innovations such as catheter-based localized drug delivery or micro-pulsed electroporation could offer more controlled and safer treatment options in such delicate anatomical areas. Regarding these complications, we propose that BEST of the aerodigestive mucosa should be performed in an interdisciplinary approach by interventional radiologists and otorhinolaryngologists. Ahead of treatment, the need for tracheostomy must be discussed with the patient, and other options to treat lesions of the laryngeal and hypopharyngeal mucosa should be explored.

Moreover, our findings support the conclusions of prior studies that BEST offers an improved adverse event profile compared to conventional sclerotherapy. Previous studies have highlighted the low risk of systemic complications associated with bleomycin injections [14,15,16]. The risk is further minimized using electroporation to enhance localized drug delivery, thereby reducing the required dose of bleomycin per lesion. McMorrow et al. demonstrated the potential of BEST to achieve significant outcomes with lower bleomycin doses, mitigating the risk of systemic toxicity such as pulmonary fibrosis [1]. The application of electroporation has been shown to enhance the vascular disruption and vascular lock effects, increasing endothelial permeability and achieving superior drug retention [17]. However, transient complications such as swelling and pain remain common, as noted in our cohort and by Kostusiak et al., who identified swelling and pain as the most frequent side effects of BEST [4].

From a diagnostic standpoint, accurate differentiation between arteriovenous, venous, lymphatic, or combined malformations and vascular tumors is essential for effective treatment planning. Differential diagnosis should be based on a comprehensive clinical assessment, imaging modalities including MRI and Doppler ultrasound, and, when necessary, histopathological evaluation. Especially in the aerodigestive tract, distinguishing vascular malformations from hemangiomas, neoplastic lesions, or post-inflammatory changes is critical to avoid inappropriate interventions and improve therapeutic outcomes.

Despite the demonstrated effectiveness of BEST, limitations remain. A key limitation of this study is its retrospective design, which inherently introduces potential biases. Additionally, the small cohort size, particularly in subgroup analyses, limits the generalizability of our findings. This is especially relevant for the hypopharyngeal and laryngeal group (*n* = 2), where severe complications, including bleeding, swelling, and the need for tracheotomy, led us to conclude that BEST is not a suitable treatment for slow-flow malformations in this region due to the high risk of serious adverse events. Therefore, no further patients with lesions in this anatomic site were treated with BEST.

Future prospective studies with larger cohorts are needed to further validate our findings and refine patient selection criteria. In particular, efforts should focus on developing predictive tools to identify patients who are most likely to benefit from BEST based on lesion type, size, and anatomical location. Combining BEST with adjunctive therapies, such as low-dose corticosteroids to reduce the inflammatory response or catheter-based delivery systems for targeted administration, may also enhance therapeutic outcomes and safety profiles. Furthermore, the variability in treatment protocols across centers highlights the need for standardized, evidence-based clinical guidelines to ensure consistent and effective application [5,17].

In addition to its effect in treating slow-flow malformations, BEST also appears to be successful in managing fast-flow lesions, as observed in a cohort of ten patients with AVMs of the cervicofacial region [18]. These findings suggest an expanded potential for BEST in the treatment landscape of vascular anomalies; however, further controlled studies are needed to determine optimal dosing strategies and long-term efficacy. Further studies are required to establish the role of BEST within the current spectrum of treatment modalities [19]. Accordingly, future research should aim to develop protocol-driven, multi-institutional studies that assess long-term outcomes and focus on functional measures such as speech, swallowing, breathing, and patient-reported quality of life.

## 5. Conclusions

Our findings demonstrate that BEST is an effective treatment for slow-flow vascular malformations of the oral cavity and tongue, with lesion volume reduction and symptom improvement. In contrast, lesions of the laryngeal and hypopharyngeal mucosa showed limited response and were associated with severe complications, including persistent dysphagia, dyspnea, and life-threatening bleeding, necessitating tracheostomy in some cases. These risks precluded further treatment in this region. Given these anatomical challenges, a multidisciplinary approach involving interventional radiologists and otorhinolaryngologists is essential.

## Figures and Tables

**Table 1:** Patient characteristics of the study cohort.

Characteristics	Cohort (Total, *n* = 20)
Age at first treatment (years), median (range)	39 (4–78)
Women	13/20
Men	7/20
Slow-flow vascular malformations of the mucosa	20/20
VMs	15/20
LMs	3/20
VLMs	2/20
Involved anatomical areas of the head and neck	
Laryngeal and hypopharyngeal mucosa	2/20
Tongue	8/20
Oral cavity	4/20
Oral cavity and tongue	6/20
Previous invasive treatments	8/20
Debulking surgery	2/20
Laser therapy	1/20
Sclerotherapy	4/20
Combination of surgery with sclerotherapy and sirolimus treatment	1/20

LM: lymphatic malformation, VLM: veno-lymphatic malformation, and VM: venous malformation.

**Table 2:** Procedural data of the study.

	Cohort (Total, *n* = 20)	BEST (Total, *n* = 29)
BESTs per patient, mean (±SD)	1.5 (±0.7)	
Total BESTs per patient		
1	13/20	
2	5/20	
3	2/20	
Primarily used electrode		
15 mm orthogonal finger electrodes		21/29
10 mm longitudinal finger electrodes		6/29
Non-divergent Stinger electrodes		2/29
Cycles of electroporation, mean (±SD)		12.7 (±13.7)
Dose (mg) of bleomycin		
Intralesional (*n* = 28), mean (±SD)		5.7 (±4.2)
Intravenous (*n* = 1)		7
Duration of procedure (minutes ± SD)		66.2 (±40.22)
Specialty performing the procedure		
Otorhinolaryngologist		6/29
Interventional Radiologist		14/29
Combined		9/29
Prophylactic anticoagulation		16/29
Surveillance in ICU after procedure		11/29
Days in ICU, mean (±SD)		5.4 (±6.4)
Days in hospital, mean (±SD)		7.0 (±7.1)

BEST: bleomycin electrosclerotherapy, ICU: intensive care unit, and SD: standard deviation.

**Table 3:** Comparison of outcomes according to the anatomic location of the lesion (*p* = 0.0318, Pearson’s chi-squared test).

Location	BEST Cohort	Lesion Reduced	Lesion Significantly Reduced	Stable Disease
Laryngeal and hypopharyngeal mucosa	2/20	1/2	0/2	1/2
Tongue	8/20	3/8	5/8	0/8
Oral cavity and tongue	6/20	1/6	5/6	0/6
Oral cavity	4/20	3/4	1/4	0/4
**Total**	20/20	8/20	11/20	1/20

BEST: bleomycin electrosclerotherapy.

**Table 4:** Comparison of functional outcomes and postinterventional symptoms according to lesion location.

Symptoms Before 1. BEST	Symptomatic Patients (*n* = 20/20)	Dysphagia	Dyspnea	Speech Impairment	Swelling	Pain	Bleeding	Infections	Cosmetic Concerns
Laryngeal and hypopharyngeal mucosa	2/20	2/2	2/2	none	none	none	none	none	none
Tongue	8/20	7/8	none	2/8	7/8	5/8	3/8	none	none
Oral cavity and tongue	6/20	4/6	1/6	2/6	5/6	5/6	2/6	2/6	none
Oral cavity	4/20	2/4	1/4	none	4/4	1/4	1/4	none	2/4
**Postinterventional symptoms** **after 1. BEST**	**Symptomatic patients** **(*n* = 17/20)**	**Dysphagia**	**Dyspnea**	**Speech impairment**	**Swelling**	**Pain**	**Bleeding**	**Infections**	**Cosmetic concerns**
Laryngeal and hypopharyngeal mucosa	2/2	2/2	2/2	1/2	1/2	none	1/2	none	none
Tongue	6/8	2/8	2/8	none	6/8	1/8	1/8	none	none
Oral cavity and tongue	6/6	2/6	none	none	5/6	none	1/6	none	none
Oral cavity	3/4	none	none	none	2/4	2/4	1/4	1/4	none
**Persistent symptoms** **after 1. BEST**	**Symptomatic patients** **(*n* = 8/20)**	**Dysphagia**	**Dyspnea**	**Speech impairment**	**Swelling**	**Pain**	**Bleeding**	**Infections**	**Cosmetic concerns**
Laryngeal and hypopharyngeal mucosa	1/2	2/2	2/2	1/2	none	none	none	none	none
Tongue	3/8	none	1/8	none	1/8	none	1/8	none	none
Oral cavity and tongue	2/6	none	none	none	2/6	none	none	none	none
Oral cavity	2/4	1/4	none	none	none	1/4	none	none	none
**Postinterventional symptoms** **after 2. BEST**	**Symptomatic patients** **(*n* = 6/7)**	**Dysphagia**	**Dyspnea**	**Speech impairment**	**Swelling**	**Pain**	**Bleeding**	**Infections**	**Cosmetic concerns**
Laryngeal and hypopharyngeal mucosa	no second treatment	-	-	-	-	-	-	-	-
Tongue	2/3	none	none	none	2/3	none	none	none	none
Oral cavity and tongue	3/3	none	none	none	2/3	1/3	none	none	none
Oral cavity	1/1	none	none	none	1/1	none	none	none	none
**Persistent symptoms** **after 2. BEST**	**Symptomatic patients** **(*n* = 1/7)**	**Dysphagia**	**Dyspnea**	**Speech impairment**	**Swelling**	**Pain**	**Bleeding**	**Infections**	**Cosmetic concerns**
Tongue	0/3	none	none	none	none	none	none	none	none
Oral cavity and tongue	1/3	1/3	none	none	none	none	none	none	none
Oral cavity	0/1	none	none	none	none	none	none	none	none
**Postinterventional symptoms** **after 3. BEST**	**Symptomatic patients** **(*n* = 1/2)**	**Dysphagia**	**Dyspnea**	**Speech impairment**	**Swelling**	**Pain**	**Bleeding**	**Infections**	**Cosmetic concerns**
Oral cavity and tongue	1/2	none	none	none	1/2	none	none	none	none
**Persistent symptoms** **after 3. BEST**	**Symptomatic patients** **(*n* = 0/2)**	none	none	none	none	none	none	none	none

BEST: bleomycin electrosclerotherapy; postinterventional symptoms are defined as those occurring during hospitalization following the intervention, whereas persistent symptoms refer to those reported by the patient during the follow-up consultation 1–3 months after treatment.

## Data Availability

The original contributions presented in this study are included in the article/Supplementary Material. Further inquiries can be directed to the corresponding author.

## Supplementary Materials

The following supporting information can be downloaded at: https://www.mdpi.com/article/10.3390/biomedicines13051055/s1, Video S1. A 59-year-old male patient with a venous malformation (VM) of the oral cavity and tongue presented with swelling. (A) Videoendoscopy of the VM at the tongue base prior to treatment. (B) Videoendoscopy 4 days after the third BEST session shows the lesion with minimal swelling and fibrin coating. Post-treatment, the patient initially experienced pain and dysphagia, which resolved within 1 week and were effectively managed with non-steroidal anti-inflammatory drugs and a soft diet. (C) 1 month after the final BEST session, the lesion demonstrated significant reduction, and the patient was completely symptom-free. Video S2. A 64-year-old female patient with a venous malformation (VM) of the laryngeal and hypopharyngeal mucosa presented with symptoms of dysphagia and dyspnea. (A) Visualization of the laryngeal and hypopharyngeal VM prior to treatment. (B) Swelling and recurrent bleeding necessitated the placement of a tracheostomy and a feeding tube for nutritional support. 21 days post-treatment, the swelling of the lesion persisted. (C) 6 months after the BEST procedure, regression of the lesion was observed, allowing for the removal of the tracheostomy tube and the resumption of oral food intake. However, the initial symptoms of dysphagia and dyspnea persisted. Due to the significant complications experienced during the first treatment, no subsequent BEST procedure was performed.

## Abbreviations

AVM	arteriovenous malformation
BEST	bleomycin electrosclerotherapy
ICU	intensive care unit
ISSVA	International Society for the Study of Vascular Anomalies
LM	lymphatic malformation
SD	standard deviation
VM	venous malformation
VLM	veno-lymphatic malformation
